# Cyclic fatigue resistance in NiTi files by four different sterilization methods

**DOI:** 10.6026/973206300220219

**Published:** 2026-01-31

**Authors:** Markapurapu Baba Sainadh, Satyam Martha, Punithavathy R, Sri Ramya M, Kondapalli Haritha, Amulya Jalumuru

**Affiliations:** 1 Department of Pediatric and Preventive Dentistry, Lenora Institute of Dental Sciences, Rajamahendravaram, Andhra Pradesh, India

**Keywords:** Pediatric endodontics, NiTi rotary files, cyclic fatigue resistance, sterilization, autoclave, sodium hypochlorite, glass bead sterilization, ethyl alcohol

## Abstract

The cyclic fatigue resistance of Pedoflex NiTi files that have been expose to four sterilization techniques. Hence, a total of fifty Pedoflex NiTi rotary instruments were selected at random and were assigned to five groups according to the type of the sterilization method used and five sterilization cycles carried out in each group. After the sterilization procedure, the evaluation of all files under cyclic fatigue resistance was done on a simulated curved canal model up to fracture. It was discovered that nickel-titanium alloy was very susceptible to thermal and mechanical stresses. Autoclave sterilization with the help of thermal treatment improved the crystallographic arrangement of the alloy and led to greater flexibility. The repeated cycles do not significantly reduce fatigue life and that autoclaving may even enhance cyclic fatigue resistance, thereby supporting evidence based decisions on safe reuse of heat treated pediatric NiTi instruments in high volume clinical settings.

## Background:

Pediatric endodontics is aimed at the treatment of pulpal and periapical diseases in primary teeth that are important in the preservation of arch integrity, healthy speech, mastication and the regulation of permanent eruption of teeth [1]. Pulpectomy is a procedure that is performed frequently in the field of pediatric endodontics, mostly implemented in the treatment of non- vital primary teeth. To a great extent the success of the pulpectomy treatment is based on the sterilization of endodontic instruments, appropriate preparation and obturation of biomechanical [2]. Biomechanical preparation of primary teeth has long been performed with stainless steel files, but because of their inherent rigidity, they are prone to cause some procedural complications, including ledging, canal transportation, zipping, and fracture of the instrument, particularly in the narrow and curved canals [3]. In their effort to overcome this limitation, NiTi rotary instruments were proposed by Walia *et al.* in 1988, which had better characteristics including increased flexibility, superelasticity and shape memory [4]. Cyclic fatigue, however, is not an issue of NiTi rotary instruments despite their high properties. Curved canals that undergo tension-compression cycling cause cyclic fatigue, which is one of the significant causes of file separation. Additionally, NiTi files can be damaged regarding their structural integrity by repeated clinical application and exposure to different sterilization and disinfection procedures. Such techniques have the capability of changing the surface properties of NiTi files resulting in great roughness, corrosion, and poor cyclic fatigue resistance [5]. Although some researchers have studied the effects of different sterilization methods and chemical agents separately on NiTi instruments, there is not much information on their interaction with each other, especially when it comes to pediatric rotary files. Taking into consideration the fact that the maintenance of the mechanical integrity of endodontic instruments is of particular importance to prevent the occurrence of procedural errors, it is necessary to assess how routine sterilization can influence the cyclic fatigue resistance of endodontic instruments [6]. Therefore, it is of interest to evaluate and compare the outcomes of various sterilization procedures and results can help facilitate the creation of more efficient and safe sterilization procedures in the treatment of children with endodontics.

## Materials and Methods:

The present *in vitro* study was conducted in the Department of Pediatric and Preventive Dentistry, Lenora Institute of Dental Sciences, Rajanagaram, Andhra Pradesh.

## Equipment used in the study:

[1] 50 - Pedoflex NiTi rotary files

[2] Steam pressure autoclave

[3] 5% NaOCl

[4] Distilled water

[5] Glass bead sterilizer

[6] 90% Ethyl alcohol

[7] Custom-made cyclic fatigue testing model

[8] Endo motor (Endoking)

[9] Stop watch

## Methodology:

Fifty Pedoflex NiTi rotary files (16 mm, 0.06 taper) were randomly divided into five groups (n=10) based on sterilization method:

[1] Group 1 (Control): No sterilization

[2] Group 2: Autoclaved at 121°C, 15 psi for 15 min

[3] Group 3: Soaked in 5% NaOCl for 30 sec, then autoclaved

[4] Group 4: Glass bead sterilization at 425°F for 10 sec

[5] Group 5: Wiped with 90% ethyl alcohol

A custom cyclic fatigue testing apparatus was designed using a stainless-steel block to simulate an artificial root canal. The canal was fabricated through precision milling and had standardized dimensions of 16 mm in length, 2 mm in width, and 2 mm in depth, with a 60 ° curvature to match the taper and geometry of the test files. The stainless-steel block included screw and hole attachments to facilitate accurate positioning of the endodontic handpiece in alignment with the artificial canal [6]. To ensure stability during testing, the handpiece was mounted on a wooden support fixture, minimizing vibrations or unintended movement. A tempered glass cover was placed over the canal to prevent file slippage and to allow visual monitoring of the file during the testing procedure. Fifty Pedoflex NiTi rotary files were divided into five groups (n = 10) based on the sterilization protocol. Group 1 (Control): Files were not subjected to any sterilization and tested directly. Group 2 (Autoclave): Files underwent five sterilization cycles at 121°C and 15 psi for 15 minutes. Group 3 (NaOCl + Autoclave): Files were immersed in 5% sodium hypochlorite for 30 seconds, rinsed, dried, and then autoclaved as in Group 2. Group 4 (Glass Bead): Files were subjected to five cycles of glass bead sterilization at 425°F for 10 seconds. Group 5 (Ethyl Alcohol): Files were wiped five times with 90% ethanol. All files were tested using a rotary motor at 350 rpm with 1.5 Ncm torque until separation occurred. Fracture time was measured using a stopwatch, and the number of cycles to fracture (NCF) was determined using the following formula [6]. NCF = (Fracture time in seconds x Rotational speed) / 60. Results were tabulated for statistical comparison among groups.

## Results:

**Figure 1:**
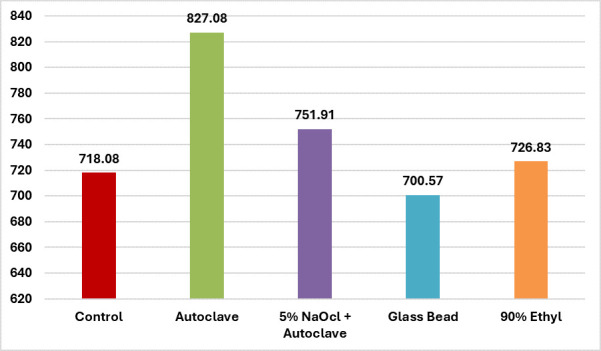
Evaluation of cyclic fatigue resistance (mean number of cycles to fracture) of NiTi files after repeated cycles of sterilization

The data obtained were input into Microsoft Excel and subsequently analyzed using SPSS software, version 23.0. Since the data exhibited a normal distribution, a one-way analysis of variance (ANOVA) was employed to compare the mean number of cycles to fracture (NCF) among the five groups. Post hoc pairwise comparisons were performed using Tukey's test. A p-value of less than 0.05 was considered statistically significant. Table 1 and Figure 1 display the mean NCF values for each group group 1 (Control) is 718.08 ± 82.92, 827.08 ± 123.01 for group 2 (Autoclave), 751.91 to ± 95.87 for group 3 (5%NaOCl + Autoclave), 700.57 ± 78.52 for group 4 (Glass bead sterilization) and 726.83 ± 115.63 for group 5 (90% Ethyl alcohol). Table 2 presents the results of the multiple group comparisons. The analysis revealed no statistically significant differences in cyclic fatigue resistance between any of the sterilization groups at the p < 0.05 level.

## Discussion:

There are various elements that influence the success of pulpectomy with effective sterilization, proper biomechanical preparation, and successful obturation being among them. The manual method of instrumentation traditionally was a means of bio-mechanical preparation that was clinically difficult in many cases. They consist of the challenges of handling young children with short attention spans and cooperation in the process of a long procedure and the high technique sensitivity when it comes to manual filing. In order to address these shortcomings, Barr *et al.* presented pediatric-specific NiTi rotary files in 2000 [7], with the advantages of: better clinical and radiographic results, less instrumentation time and less postoperative pain. Nevertheless, the issue of fracture of the instrument during the biomechanical preparation is a significant one and may lead to the poor treatment outcomes. According to Sattapan *et al.* (2000), the two main failure modes in NiTi rotary instruments torsional fracture and cyclic fatigue were found. Torsional fracture is an effect of the tip or part of the file becoming stuck in the canal as the handpiece keeps rotating, usually as a result of the high apical pressure this is particularly noticeable in smaller-diameter files, and is most often visibly marked by unraveling or bending of the flutes [8]. Pedulla *et al.* (2022) further subdivided torsional failure into static and dynamic torsional failure, in which the former occurs when the file suddenly locks-in, whereas the latter occurs gradually as a result of accumulation of friction when the instrument passes through the dentin [9]. On the contrary, cyclic fatigue is caused by repeated actions of tension and compression especially when the instrument is used in free rotation in curved canals. These is a mechanical stress and stress that results in the formation of micro cracks and fracture, normally without any noticeable deformation. Fatigue in instruments of large sizes is more common in cyclic form, and this is a major issue in clinical endodontics. The thermal, manufacturing and finishing methods like electropolishing and post-machining heat treatment of nickel- titanium (NiTi) play a big role in making it adaptable and robust in endodontics [8]. Although the fracture rate of NiTi rotary files has been comparatively low because of the development of alloy design, heat treatment and manufacturing methods, even in normal pediatric endodontics, use of NiTi rotary files may be influenced by clinical outcomes. Disposing NiTi files at the conclusion of single use is not always practical in high-volume pediatric clinical and public health environments due to financial constraints. These instruments are therefore usually reused and their sterilization cycles repeated to ensure that there is an absence of infection. Nevertheless, frequent exposure to high temperature sterilization like autoclaving and glass bead sterilization or chemical treatment using sodium hypochlorite (NaOCl) and ethyl alcohol may degrade the surface of the files by corrosion and roughness [10].

The above-discussed sterilization procedures can also be the cause of file fracture, hence, in this research the autoclave, 5% NaOCl with autoclave, glass bead sterilizer and ethyl alcohol have been chosen as the most common ways of sterilization and disinfection. The most common method of sterilization used in dentistry is autoclaving because it is the most effective in destroying pathogen, however repetitive cycles may change the microstructure and reduce the cyclic fatigue strength of non-heat-treated NiTi files [11]. Autoclaving with 5 percent sodium hypochlorite gives a complete disinfection effect and can also lead to corrosion of the surfaces [12]. Although glass beads are convenient in chairside sterilization, they expose instruments to extreme high temperatures of dry heat which might affect the structural integrity of instruments [13]. Ethyl alcohol (90-year old) is a fast disinfectant, which is non-sterile and may not fully sterilize the surface of NiTi files when used many times [12]. Recently there have also been innovations on pediatric endodontics (Pedoflex file system) in which the heat-treated technology is introduced, which demonstrates to be promising and more efficient in cleaning the root canals with the alleged advantage of less invasive and faster treatment. They also exhibited greater cyclic fatigue resistance that is another added feature of these files systems [13]. The knowledge of the effects of sterilization is essential in assessing the clinical effect of longevity and safety of NiTi instruments particularly in cyclic fatigue conditions that are usually experienced in curved canals. Thus, the current research aims at assessing and comparing the impact of five repetitive cycles of four various sterilization techniques on cyclic fatigue resistance of 50 Pedoflex NiTi files of 16mm length and 0.06 taper were separated into five groups according to the sterilization method. A cyclic fatigue testing model was designed and custom made with a length of 16 mm, width of 2 mm and depth of 2 mm with 60 radius of curvature to simulate the mechanical loads that are exerted at the curved root canals, specifically in pediatric molars. This 60 degree arc also displays moderate-severe curved canals that are typical mostly in the primary teeth as well as the immature permanent teeth [7]. All the files were inserted into an artificial canal of 60 o curvature and the rotation was performed at a constant speed of 350 rpm, torque of 1.5 N cm with contra angled hand piece. The files were rotated until the fracture of the file was achieved, time taken to complete this was noted down with the help of stopwatch. All the data were summarized and statistically analyzed. Table 1 shows the average fracturing cycle, which is a measure of cyclic fatigue strength of NiTi files following five sterilization cycles. The outcome indicated that there were no significant differences in the groups (p < 0.05). Group 1 (Control) indicated low means of cyclic fatigue resistance at 718.08 /82.92 when compared to group 2 (Autoclave), group 3 (5 Percent of NaOCl followed by autoclave) and group 5 (90 Percent Ethyl alcohol) and high when compared to group 4 (Glass bead sterilization). These unspent stresses may serve as a weakness point, which facilitates microcrack formation and propagation with repetitive stress cycles. Unless relieved by heat treatment, these stresses may decrease the fatigue life of NiTi instruments. The control samples were not sterilized and thus it is possible that these pre-existing stresses were not altered to cause the lower resistance [14]. Group 2 files i.e., (Autoclave) were found to have the greatest mean cyclic fatigue resistance (827.08 ± 123.01) compared to group 1 (Control), group 3 (5% NaOCl followed by autoclave), group 4 (Glass bead sterilization) and group 5 (90% Ethyl alcohol) files. Since repetitive autoclaving can cause the alloys of NiTi to experience stress relief or phase stability following thermal treatment, it increases the fatigue strength. Al-Amidi et al. found that repeated autoclave cycles did not adversely affect the cyclic fatigue resistance of several heat treated NiTi rotary systems and, for some files, even increased the number of cycles to fracture [15]. The cyclic fatigue resistance increased statistically significantly in ProFile NiTi files which had undergone five cycles of autoclave sterilization. This was enhanced by the fact that it could have been a stress reliever and some microstructural changes produced by thermal exposure that increased the flexibility and fatigue strength of the alloy [14]. In tandem with these observations, Guttman *et al.* (2012) found that there were high numbers of cycles to failure after autoclaving, which was recommended to be caused by rejuvenation of the NiTi alloy by the heat treatment [16]. Similarly, Shaik *et al.* (2024) demonstrated that repeated sterilization cycles and irrigant exposure induce nanostructural surface changes and increased roughness on NiTi rotary instruments, potentially predisposing them to reduced cyclic fatigue resistance and earlier fracture [17]. Those by Hifler *et al.* (2011) also said that cyclic fatigue resistance decline following autoclaving [18]. Group 3 (5% NaOCl and autoclave) showed more cyclic fatigue resistance (751.91 + 95.87) in comparison to group 1 (Control), group 4 (Glass bead sterilization) and group 5 (90% Ethyl alcohol) but lower cyclic fatigue resistance in comparison to group 2 (Autoclave). Contrarily, our study Dioguardi *et al.* (2021) [19] has found that the resistance to cyclic fatigue of NiTi files in glass bead sterilization is enhanced. Group 5 (90% Ethyl alcohol) exhibited higher cyclic fatigue resistance (726.83 115.3) than group 1 (Control), and group 4 (Glass bead sterilization), and lower fatigue resistance than group 2 (Autoclave) and group 3 (5% NaOCl followed by autoclave). This shows that there was no significant difference in cyclic fatigue resistance of NiTi files with ethanol sterilization. Ethyl alcohol as a chemical disinfectant does not result to full sterilization that may indirectly influence cyclic fatigue resistance in a series of sterilization processes particularly when microbial remain in the instruments causing localized corrosion or surface imperfections on instruments [20]. The present study statistical analysis indicated that inter group comparison with non-significant difference with p value less than 0.05 was observed, whereas group 2 (autoclave) enhanced the cyclic fatigue resistance of NiTi files and this was found to be less than the other groups (including control, 5% NaOCl + autoclave, glass bead sterilization, and ethyl alcohol). Nickel-titanium (NiTi) is very susceptible to thermal and mechanical stress. Autoclave sterilization by thermal treatment has been reported to increase the crystallographic order of the alloy to give higher flexibility. Also, it can cause phase changes which lead to higher resistance to cyclic fatigue fracture. There were however some limitations. The experimental design is not able to recreate the biological, thermal, and mechanical conditions of paediatric root canals. The experiment only addressed fatigue resistance in cyclic mode; other qualities like torsional strength, flexibility and cutting efficiency were not addressed. SEM and associated techniques were not used to examine surface and structural changes.

## Conclusion:

Each of the sterilization means used in this experiment exhibited a certain effect on the cyclic fatigue resistance but none of them showed a significant statistical difference. The results justify the application of autoclaving as the sterilization procedure of pedoflex NiTi files as the standard. Further validation is needed with large sample size and long term clinical observation.

## Figures and Tables

**Table 1:** Evaluation of cyclic fatigue resistance (mean number of cycles to fracture) of NiTi files after repeated cycles of sterilization.

**TEST GROUPS (n=10)**	**MEAN**	**STANDARD DEVIATION**	**p VALUE**
Control	718.08	82.92	0.06 NS
Autoclave	827.08	123.01	
5%NaOCl + Autoclave	751.91	95.87	
Glass bead sterilization	700.57	78.52	
90% Ethyl alcohol	726.83	115.63	

**Table 2:** Mean multiple group comparison of cyclic fatigue resistance of NiTi files after repeated cycles of sterilization.

**TEST GROUPS**	**MEAN**	**STANDARD DEVIATION**
Control - Autoclave	-109	.129 NS
Control - 5%NaOCl + Autoclave	-33.83	.943 NS
Control - Glass bead sterilization	17.5	1.000 NS
Control - 90% Ethyl alcohol	-8.75	.995 NS
Autoclave - 5%NaOCl + Autoclave	75.16	.463 NS
Autoclave - Glass bead sterilization	126.5	.189 NS
Autoclave - 90% Ethyl alcohol	100.25	.054 NS
5%NaOCl + Autoclave - Glass bead sterilization	51.33	.981 NS
5%NaOCl + Autoclave - 90% Ethyl alcohol	25.08	.785 NS
Glass bead sterilization - 90% Ethyl alcohol	-26.25	.977 NS
